# The red blood cell as a mediator of endothelial dysfunction in patients with familial hypercholesterolemia and dyslipidemia

**DOI:** 10.1111/joim.13580

**Published:** 2022-11-02

**Authors:** Ali Mahdi, Tigist Wodaje, Oskar Kövamees, John Tengbom, Allan Zhao, Tong Jiao, Marcus Henricsson, Jiangning Yang, Zhichao Zhou, Anni I. Nieminen, Malin Levin, Aida Collado, Jonas Brinck, John Pernow

**Affiliations:** ^1^ Division of Cardiology Department of Medicine Solna Karolinska Institutet Karolinska University Hospital Stockholm Sweden; ^2^ Division of Cardiology Department of Medicine Huddinge Karolinska Institutet Karolinska University Hospital Stockholm Sweden; ^3^ Department of Physiology and Pharmacology Karolinska Institutet Stockholm Sweden; ^4^ Department of Molecular and Clinical Medicine Wallenberg Laboratory Institute of Medicine The Sahlgrenska Academy at University of Gothenburg and Sahlgrenska University Hospital Gothenburg Sweden; ^5^ FIMM Metabolomics Unit Institute for Molecular Medicine Finland University of Helsinki Helsinki Finland; ^6^ Division of Endocrinology Department of Medicine Huddinge Karolinska Institutet Karolinska University Hospital Stockholm Sweden

**Keywords:** arginase, endothelial dysfunction, familial hypercholesterolemia, low‐density lipoprotein, reactive oxygen species, red blood cell

## Abstract

**Background:**

Patients with familial hypercholesterolemia (FH) display high levels of low‐density lipoprotein cholesterol (LDL‐c), endothelial dysfunction, and increased risk of premature atherosclerosis. We have previously shown that red blood cells (RBCs) from patients with type 2 diabetes induce endothelial dysfunction through increased arginase 1 and reactive oxygen species (ROS).

**Objective:**

To test the hypothesis that RBCs from patients with FH (FH‐RBCs) and elevated LDL‐c induce endothelial dysfunction.

**Methods and results:**

FH‐RBCs and LDL‐c >5.0 mM induced endothelial dysfunction following 18‐h incubation with isolated aortic rings from healthy rats compared to FH‐RBCs and LDL‐c <2.5 mM or RBCs from healthy subjects (H‐RBCs). Inhibition of vascular but not RBC arginase attenuated the degree of endothelial dysfunction induced by FH‐RBCs and LDL‐c >5.0 mM. Furthermore, arginase 1 but not arginase 2 was elevated in the vasculature of aortic segments after incubation with FH‐RBCs and LDL‐c >5.0 mM. A superoxide scavenger, present throughout the 18‐h incubation, attenuated the degree of endothelial dysfunction induced by FH‐RBCs and LDL‐c >5.0 mM. ROS production was elevated in these RBCs in comparison with H‐RBCs. Scavenging of vascular ROS through various antioxidants also attenuated the degree of endothelial dysfunction induced by FH‐RBCs and LDL‐c >5.0 mM. This was corroborated by an increase in the lipid peroxidation product 4‐hydroxynonenal. Lipidomic analysis of RBC lysates did not reveal any significant changes across the groups.

**Conclusion:**

FH‐RBCs induce endothelial dysfunction dependent on LDL‐c levels via arginase 1 and ROS‐dependent mechanisms.

## Graphical abstract

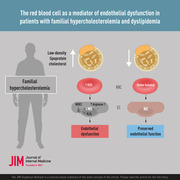

## Introduction

Atherosclerotic cardiovascular disease (CVD) and its clinical manifestations—myocardial infarction, peripheral artery disease, and stroke—are widely recognized as multifactorial with contributions from both lifestyle and genetic factors. Among several risk factors, elevated cholesterol levels have been established as a key risk factor for premature myocardial infarction [1]. Patients with familial hypercholesterolemia (FH) have a genetic predisposition for CVD due to mutations mainly in the low‐density lipoprotein (LDL) receptor with premature atherosclerosis if left untreated [2, 3]. The mechanisms underlying the development of atherosclerotic lesions remain incompletely understood. An early event is the development of dysfunction of the endothelial layer with subsequent retention and oxidation of LDL‐cholesterol (LDL‐c), which attracts inflammatory cells to the lesion. In patients with FH, this process is accelerated and aggravated due to an excess of LDL particles [4]. However, the mechanisms initiating endothelial dysfunction in hypercholesterolemia have not been fully identified.

The red blood cell (RBC) has emerged as an important mediator of aggravated cardiovascular injury owing to its ability to modulate vascular homeostasis through an imbalance between oxidative metabolites and its antioxidative properties [5, 6]. The structure and function of RBCs are affected by circulating levels of plasma LDL‐c. It has been shown that cholesterol is accumulated in the RBC plasma membrane, causing a decrease in deformability and increased stiffness [7]. Also, RBC‐derived cholesterol contributes to the progression of the atherosclerotic plaque, supported by the observation that total cholesterol is higher in membranes of RBCs from patients with vulnerable plaques than in patients with stable plaques [8].

We have recently shown that an altered property of RBCs in patients with type 2 diabetes and COVID‐19 induces endothelial dysfunction [9, 10, 11]. This effect induced by RBCs involves upregulation of the enzyme arginase 1, which competes with the endothelial nitric oxide synthase (eNOS) for their common substrate L‐arginine. In situations of increased arginase activity, the limited availability of L‐arginine for eNOS results not only in decreased nitric oxide production but also in uncoupling of eNOS with an increase in the formation of reactive oxygen species (ROS), most notably superoxide [12]. Consequently, we were able to observe an increase in ROS formation in RBCs from type 2 diabetes and COVID‐19 patients, which contributes to endothelial injury [9, 10]. These novel results indicate that the RBC is an important mediator of vascular injury in the presence of cardiovascular risk factors.

Based on the above, we tested the hypothesis that RBCs from patients with FH (FH‐RBCs) exert a negative effect on endothelial function and that such an effect is mediated through changes in the redox balance. As the levels of circulating cholesterol affect the lipid content in RBCs, we further explored the influence of LDL‐c levels on RBC‐induced endothelial dysfunction. We observed that FH‐RBCs and high levels of LDL‐c (LDL >5.0 mM) but not low LDL‐c levels (LDL <2.5 mM) impaired endothelial function.

## Material and methods

### Study subjects

Thirty‐six patients with FH were recruited from the outpatient clinic of the Department of Diabetology and Endocrinology of Karolinska University Hospital between 2016 and 2021. The diagnosis of FH was based on a Dutch Lipid Clinic Network Score of more than 6. To assess the influence of LDL‐c, two groups of FH patients were included: one group with LDL‐c >5.0 mM (*n* = 29) and one group with LDL‐c <2.5 mM (*n* = 10). Exclusion criteria were coronary artery diseases, previous stroke, peripheral artery disease, diabetes mellitus (type 1 or 2), excessive alcohol consumption, or age <18 years. A total of 24 healthy subjects without a history of CVD were recruited among subjects who previously had participated in studies at the Department of Cardiology, Karolinska University Hospital, Solna, Sweden. Routine blood clinical chemistry values and hemodynamic parameters were recorded on the day of participation or extracted from patient charts. Participants were informed about the purpose and possible risks of the study, and all gave their oral and written consent. The investigation was approved by the Swedish Ethical Review Authority and conducted according to the Declaration of Helsinki.

### Animals

Male wild‐type Sprague–Dawley or Wistar rats at the age of 9–16 weeks (Charles River, Sulzfeld, Germany) were anesthetized with pentobarbital (50 mg/kg i.p.) followed by thoracotomy and isolation of the aortas. Aortas were cleaned from adipose and connective tissue, and segments were cut into 2 mm segments and incubated with RBCs for the determination of endothelial function as described below [9]. Animal care and all protocols were approved by the regional ethical committee (17708‐2019) and conformed to the Guide for Care and Use of Laboratory Animals published by the US National Institutes of Health (NIH publication no. 85‐23, revised 1996).

### RBC incubations and vessel reactivity experiments

Venous blood samples were collected in heparinized tubes from patients with FH and healthy controls following an overnight fasting period. Isolation of RBCs was performed immediately, as previously described [9]. In brief, tubes were centrifuged at 1000 g and 4°C for 10 min and subsequently washed three times with Krebs–Henseleit (KH) buffer, resulting in the successful removal of >98% of platelets and >99% of leukocytes [13]. Isolated RBCs were diluted to a hematocrit of ∼45% with KH buffer and incubated for 18 h with aortic segments in the absence and presence of the arginase inhibitor 2(S)‐amino‐6‐boronohexanoic acid (ABH, 10 mM) or the superoxide dismutase mimetic 4‐hydroxy‐2,2,6,6‐tetramethylpiperidine‐N‐oxyl (TEMPOL, 10 mM) to assess the influence of arginase and superoxide. Segments were then thoroughly washed and mounted on a wire myograph (Danish Myo Technology A/S, Hinnerup, Denmark). Endothelium‐dependent relaxation (EDR) was assessed through the application of acetylcholine (10^−9^–10^−5^ M) to vascular segments preconstricted with phenylephrine (1–10 μM) alone or in combination with 9,11‐dideoxy‐11α,9α‐epoxymethanoprostaglandin F2α (U46619, 10–30 nM) in order to achieve a stable preconstriction. Endothelium‐independent relaxation (EIDR) was assessed by the application of sodium nitroprusside (10^−9^–10^−5^ M). To further characterize the influence of FH‐RBCs and LDL‐c >5.0 mM on the vasculature, separate experiments were conducted with incubation of the aortic segment with ABH (100 μM), TEMPOL (100 μM), the hydrogen peroxide catalyst catalase (200 U/ml), the NADPH oxidase (NOX)1/2 inhibitor apocynin (100 μM), the NOX1 inhibitor 2‐acetylphenothiazine (ML‐171, 1 μM), and the NOX2 inhibitor *N*‐(1‐isopropyl‐3‐(1‐methylindolin‐6‐yl)‐1H‐pyrrolo[2,3‐b]pyridin‐4‐yl)‐1‐methyl‐1H‐pyrazole‐3‐sulfonamide (GSK2795039, 10 μM) for 1 h following the co‐incubation of RBCs and aortic segments and removal of RBCs but prior to evaluation of EDR and EIDR. We have previously shown that there is no carry‐over effect by the arginase inhibitor or ROS scavengers in these experimental protocols and that there is no effect of arginase inhibition, ROS scavenging, or NOX inhibition on EDR following 18‐h incubation with RBCs from healthy subjects (H‐RBCs) [9].

## Arginase activity assay

RBCs were lysed with radioimmunoprecipitation assay (RIPA) lysis buffer (Amresco, Solon, OH) containing protease inhibitors (Roche, Mannheim, Germany). Arginase activity was determined by a colorimetric assay as previously described [9]. Briefly, arginase was activated by the addition of MnCl_2_ and incubated for 10 min at 56°C. Excessive amounts of L‐arginine (0.5 M) were added and incubated at 37°C (30 min) to catalyze the reaction. The reaction was then stopped by a solution containing H_2_SO_4_:H_3_PO_4_:H_2_O. α‐Isonitrosopropiophenone, which reacts with urea and thereby generates a colored product, was added. The color intensity was quantified by a spectrophotometer (Wallac 1420 VICTOR2^TM^, PerkinElmer, Waltham, MA) at a wavelength of 540 nm. Arginase activity was calculated as urea production (mmol/mg protein/min) and expressed as percentage of control.

### Immunoblotting

Immunoblotting was performed as previously described [9]. Briefly, protein extracts from vessel rings incubated with RBCs were lysed with RIPA buffer and homogenized. Total protein content was quantified by a bicinchoninic protein assay kit (Pierce Technology, Life Technologies, Carlsbad, CA). Equal amounts of protein were separated on 10% SDS gel and transferred onto 0.45 μm nitrocellulose‐blotting membranes (Amersham, GE Healthcare, Chicago, IL). Following blocking with 5% milk for 1 h at room temperature, membranes were incubated overnight at 4°C with primary antibodies for arginase 1 (1:1000 dilution, catalog No. HPA003595; Atlas Prestige Antibody, Sigma‐Aldrich, St. Louis, MO) and GAPDH (1:2500 dilution, catalog No. G9545; Atlas Prestige Antibody, Sigma‐Aldrich) followed by incubation of a secondary antibody (IRDye® 800CW goat anti‐rabbit, 1:20000 dilution, catalog No. 926‐32211; LI‐COR Biosciences, Lincoln, NE) for 1 h at room temperature. Band densities were analyzed with Image Studio Lite Version 3.1 (LI‐COR Biosciences), normalized to GAPDH, and presented as the ratio of arginase 1/GAPDH.

### Immunohistochemistry

Following incubation with RBCs for 18 h, rat aortic rings were fixed for 24 h in 4% formaldehyde at room temperature, dehydrated in graded ethanol (70%, 95%, and 99%), embedded in paraffin, sectioned using a microtome, and mounted on coated glass slides (Superfrost® plus; Thermo Fisher Scientific, Waltham, MA) as previously described [10]. At least six slides, containing ∼4 tissue cross‐sections (5‐μm thick) from each animal were examined. Sections were deparaffinized in xylene and rehydrated in graded ethanol. For antigen retrieval, slides were subjected to high‐pressure boiling in citrate buffer (pH 6.0). After peroxidase inactivation (0.3%) and blockade with goat serum (Abcam, Cambridge, UK), aorta cross‐sections were incubated overnight (4°C) with the following primary antibodies: a rabbit polyclonal antihuman arginase 1 (1:100 dilution, catalog No. HPA003595; Atlas Prestige Antibody, Sigma‐Aldrich), a rabbit polyclonal antihuman arginase 2 (1:50 dilution, catalog No. HPA000663; Atlas Prestige Antibody, Sigma‐Aldrich), or a mouse monoclonal anti‐4‐hydroxynonenal (4‐HNE, 1:100 dilution, IgG_2B_, catalog No. MAB3249; R&D Systems, Inc., Minneapolis, MN). Specific labeling was detected using a labeled horseradish peroxidase (HRP) polymer conjugate as a secondary antibody as part of the EnVision^+^ Dual Link System‐HRP (Dako, Agilent Technologies, Santa Clara, CA). To confirm the specificity of antibodies, isotype controls were used as negative controls (rabbit IgG or mouse IgG_2B_, both from Abcam). Samples were developed using a solution containing 3, 3’‐diaminobenzidine (Dako), then counterstained with Mayer's Modified Hematoxylin (Abcam) and mounted in a mounting medium (Abcam). Fields from each aortic section were captured (Leica DM3000 Digital microscope; Leica Biosystems, Wetzlar, Germany), digitized, and analyzed (*ImageJ *software 1.52v, Bethesda, MA). Adventitial layers were not included for quantification.

### Electron spin resonance

ROS formation in RBCs was determined using electron spin resonance (ESR) as previously described [9, 14]. Briefly, washed RBCs were diluted to a hematocrit of 1% with Krebs/HEPES buffer. The RBCs were incubated with 1‐hydroxy‐3‐methoxycarbonyl‐2,2,5,5‐tetramethylpyrrolidine (200 μM) in the presence of 25 μM deferoxamine and 5 μM diethyldithiocarbamate (Noxygen Science Transfer & Diagnostics GmbH, Elzach, Germany) for 30 min at 37°C and 21% O_2_ with/without ABH (1 mM) or *N* ^ω^‐nitro‐L‐arginine methyl ester (L‐NAME, 100 μM). The cell suspensions were then frozen in liquid nitrogen and stored at −80°C until analyses. ROS formation was detected by ESR using the following settings: center field 1.99 g, microwave power 1 mW, modulation amplitude 9 G, sweep time 10 s, number of scans 10, and field sweep 60 G. The amount of CM• was determined from the calibration using 3‐carboxy‐proxyl (CP•, Noxygen Science Transfer & Diagnostics GmbH).

### Lipidomic analyses in RBCs lysates

Quantitative lipidomics of RBC lysates from three groups was performed using ultrapressure liquid chromatography (UPLC) coupled to a quadrupole ion trap (QTRAP) mass spectrometer [15] in collaboration with the Institute for Molecular Medicine Finland. Lipids were extracted from cell lysates with the liquid–liquid extraction method using ethylacetate and methanol (MeOH). First, 1 ml of liquid chromatography‐mass spectrometry (LC‐MS) grade water was added to the cell samples into the tubes, followed by three cycles for sonication and vortexing. Subsequently, the mixture was transferred into glass tubes with 50 μl of labeled internal standard mixture (SCIEX), 3.5 ml ethylacetate, 1 ml of MeOH, and 1 ml of water, followed by rotation at 30 rpm for 15 min. Samples were centrifuged for 10 min at 3000 rpm at 4°C, and the 3 ml of upper layer was collected in Borosilicate Glass Tubes and dried under N_2_ gas. Finally, the dried samples were reconstituted with 250 μl of mobile phase (dichloromethane:methanol [50:50] containing 10 mM ammonium acetate). Fifty microliters of samples were directly injected with a mobile phase at a flow rate of 70 μl/min and lipid separation and quantitation was performed with Lipidyzer™ platform using a SCIEX 5500 QTRAP® mass spectrometer (SCIEX, Washington, DC, USA) with SelexION® Differential ion mobility technology with two acquisition methods, with and without SelexION® technology, and multiple reaction monitoring (MRM) strategy with positive and negative polarities. Lipidomics Workfow Manager software was used for acquisition of samples, automated data processing, signal detection, and lipid species concentration (mM) calculations. Lipid species and subspecies were annotated according to their molecular composition. Data were then normalized as nmol/500 million cells and log transformed. Data were then processed in the R programming language (version 4.2.0). Missing values were then imputed using the k‐Nearest Neighbor imputation method. The resulting data were subsequently analyzed and visualized using the LipidR pipeline, with *p* <0.05 as the level for statistical significance [16]. The untargeted lipidomics data were mapped to 383 lipids (cholesteryl ester, ceramide, diacylglycerol, dihydroceramide [DCER], free fatty acids, hexosylceramide, actosylceramide, lysophosphatidylcholine, lysophosphatidylethanolamine [LPE], phosphatidylcholine [PC], phosphatidylethanolamine [PE], sphingomyelin [SM], and triacylglyceride) from 38 patients. Data from 244 different lipids were left after application of a 66% occupational threshold, as described previously [17], meaning that we only considered lipids that occurred in at least 66% of the total samples.

### Quantification of oxysterols

Oxysterols from RBC lysates were extracted using methanol and methyl‐tert‐butyl ether (MTBE). In brief, 10 μl of RBC lysate was added to 290 μl of water containing 120 mM ammonium acetate and 2 mM of EDTA. After a gentle mix, 300 μl of methanol (containing deuterated internal standards) was added and the samples were vortexed for 5 min at 1400 rpm. Then 1000 μl of MTBE was added and after vortexing for 5 min at 1400 rpm, the samples were spun at 500 g for phase separation. The top layer was evaporated, and oxysterols were derivatized to picolinyl esters as previously described [18]. The analysis was performed using UPLC coupled to a SCIEX QTRAP 5500 operated in positive MRM mode. Separation of the derivatized oxysterols was made using a Kinetex C18 column (Phenomenex, 2.1 × 100 mm, 1.7 μm particles) kept at 60°C with mobile phases consisting of water and acetonitrile, both containing 0.1% formic acid. Quantification was made against an external standard curve. All reference substances were attained from Avanti Polar Lipids (Alabaster, AL).

### Statistical analyses

Concentration–response curves were analyzed with stacked two‐way analysis of variance (ANOVA) with Tukey's post‐hoc test if more than two groups were compared, and significance was calculated for all the concentration–response curves. To assess the influence of a drug, two‐way ANOVA matching for both concentration and relaxation was performed, as these data are paired. Comparisons between two groups were performed with Student's *t*‐test or Mann–Whitney test, depending on normality. Differences between more than two groups were performed with one‐way ANOVA with Dunnett's, Dunn's, or Tukey's post‐hoc test. Normality was checked with D'Agostino Person test. Data are presented as means ± standard error of the mean or boxplots and min.–max. for experimental data and means ± standard deviation or median and Q1–Q3 (depending on distribution) for clinical characteristics. Lipidomic analyses were performed as described separately above. Two‐sided *p* <0.05 was considered statistically significant. All analyses were performed with GraphPad Prism v.7.02 (GraphPad Software, Inc., La Jolla, CA) apart from the lipidomic analyses, which was performed in the R programming language, as described above and previously [16].

## Results

### Study subjects

Characteristics of the study subjects are presented in Table 1. The group of FH patients with LDL‐c >5.0 mM was slightly younger, and the proportion of females was higher compared to the healthy group. The subjects had similar body mass index, hemodynamic parameters, glycemic indices, and erythrocyte indices. Triglyceride and high‐density lipoprotein levels were similar across the three groups. By design, the levels of total cholesterol and LDL‐c were markedly higher in the FH group with LDL‐c >5.0 mM than among the healthy subjects and FH patients with LDL‐c <2.5 mM, confirming the adherence to our inclusion criteria and allocation. Almost half of the subjects in the FH group with LDL‐c >5.0 mM and all subjects in the LDL‐c <2.5 mM group were on lipid‐lowering treatment.

**Table 1:** Demographics and laboratory parameters of the study subjects

	Healthy, *n* = 24	FH LDL >5.0 mM, *n* = 29	FH LDL <2.5 mM, *n* = 10	Statistical test
Age (years)	56 ± 15	45 ± 17*	57 ± 6	One‐way ANOVA^a^
Sex, male, *n* (%)	19 (79)	12 (41)	6 (60)	Chi‐square test*
Smokers, *n*	0	3	0	Chi‐square test
BMI, kg/m^2^	25 ± 3	26 ± 4	28 ± 3	One‐way ANOVA^a^
SBP, mmHg	133 ± 13	124 ± 19	132 ± 11	One‐way ANOVA^a^
DBP, mmHg	84 ± 7	80 ± 11	82 ± 7	One‐way ANOVA^a^
Fasting plasma glucose, mM	5.7 (5.4–5.8)	5.4 (5.1–5.6)	5.5 (5.4–6.0)	Kruskal–Wallis test^b^
HbA1c, mmol/mol	35 ± 3	35 ± 3	37 ± 3	One‐way ANOVA^a^
Hemoglobin, g/L	146 (136–154)	140 (132–147)	143 (136–150)	Kruskal–Wallis test^b^
RBC count, 10^12^/L	4.8 (4.5–5.0)	4.8 (4.4–5.0)	4.7 (4.4–5.1)	Kruskal–Wallis test^b^
EVF	0.43 (0.41–0.44)	0.42 (0.39–0.43)	0.43 (0.41–0.44)	Kruskal–Wallis test^b^
MCV, fL	89 ± 4	89 ± 4	90 ± 5	One‐way ANOVA^a^
MCH, pg	30 ± 2	30 ± 1	30 ± 1	One‐way ANOVA^a^
Creatinine, ug/L	79 (70–84)	77 (67–87)	72 (68–86)	Kruskal–Wallis test^b^
Triglycerides, mmol/L	0.9 (0.7–1.6)	1.2 (0.9–1.6)	1.4 (0.9–2.0)	Kruskal–Wallis test^b^
Cholesterol, mmol/L	5.2 (4.5–6.0)	8.6 (7.8–10.2)**	3.5 (2.9–4.4)^*****^	Kruskal–Wallis test^b^
HDL, mmol/L	1.6 ± 0.4	1.5 ± 0.5	1.6 ± 0.5	One‐way ANOVA^a^
LDL, mmol/L	3.0 (2.5–3.7)	6.5 (5.8–8.1)**	1.4 (1.0–2.1)^*****^	Kruskal–Wallis test^b^
Apolipoprotein A1, g/L	‐	1.5 (1.3–1.7)	1.5 (1.3–1.7)	Student´s *t*‐test
Apolipoprotein B, g/L	‐	1.8 (1.5–2.1)	0.7 (0.4–1.3)^****^	Mann–Whitney test
Medication, *n* (%)				
Statin	‐	13 (45)	8 (80)	Fisher's exact test
Ezetemib	‐	9 (31)	7 (70)	Fisher's exact test
PCSK9i	‐	3 (10)	2 (20)	Fisher's exact test
ACEi/ARB	‐	2 (7)	4 (40)^***^	Fisher's exact test
B blocker	‐	4 (14)	0 (0)	Fisher's exact test
Combination of LL drugs				
0	‐	13 (45)	0 (0)^***^	Fisher's exact test
1	‐	8 (28)	4 (40)	Fisher's exact test
2	‐	8 (28)	6 (60)	Fisher's exact test
3	‐	0 (0)	0 (0)	Fisher's exact test

*Note*: Values are mean ± standard deviation, or count, *n* (%).

Abbreviations: ACEi, angiotensin‐converting enzyme inhibitor; ARB, angiotensin receptor blocker; BMI, body mass index; DBP, diastolic blood pressure; EVF, erythrocyte volume fraction; FH, familial hypercholesterolemia; HbA1c, hemoglobin A 1c; HDL, high‐density lipoprotein; LDL, low‐density lipoprotein; LL, lipid lowering; MCH, mean corpuscular hemoglobin; MCV, mean corpuscular volume; PCSK9i, proprotein convertase subtilisin/kexin type 9 inhibitor; RBC, red blood cell; SBP, systolic blood pressure.

^a^ With Holm–Sidak's multiple comparison.

^b^ With Dunn's multiple comparison.

* *p* < 0.05 versus healthy or across groups.

** *p* < 0.001 versus healthy.

*** *p* < 0.05 versus FH LDL >5.0 mM.

**** *p* < 0.01 versus FH LDL >5.0 mM.

***** *p* < 0.001 versus FH LDL >5.0 mM.

### FH‐RBCs and dyslipidemia induce endothelial dysfunction

**Fig. 1:**
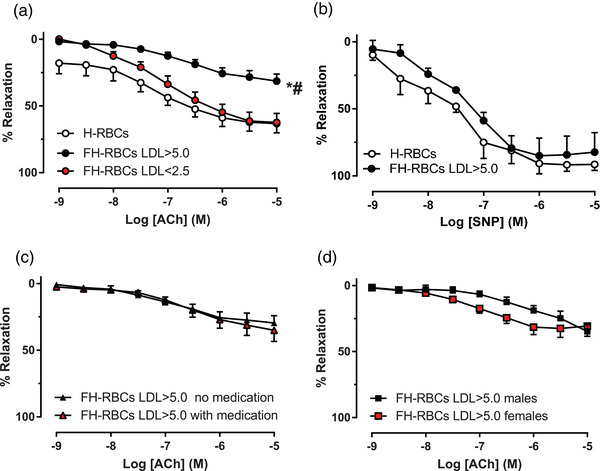
(a) Endothelium‐dependent relaxation (EDR) in aortic rings evoked with acetylcholine (ACh) following 18‐h incubation with red blood cells from healthy subjects (H‐RBCs, n = 6) and patients with familial hypercholesteremia and low‐density lipoprotein cholesterol levels >5.0 mM (FH‐RBCs LDL >5.0, n = 22) and <2.5 mM (FH‐RBCs LDL<2.5, n = 7). (b) Endothelium‐independent relaxation in aortic rings evoked with sodium nitroprusside (SNP) following incubation with H‐RBCs (n = 4) and FH‐RBCs LDL>5.0 (n = 4). (c) EDR following 18‐h incubation with FH‐RBCs LDL>5.0 from patients without (no medication, n = 11) and with lipid‐lowering treatment (with medication, n = 11) or (d) subdivided into males (n = 9) and females (n = 13). *p <0.001 versus H‐RBCs; **p <0.01 versus FH‐RBCs LDL <2.5, with two‐way analysis of variance with (a) or without (b–d) Tukey's post‐hoc test. Data are presented as the mean and standard error of means.

First, we tested the hypothesis that FH‐RBCs impair endothelial function depending on LDL‐c levels. FH‐RBCs and high LDL‐c (>5.0 mM) impaired EDR in aortic segments compared to H‐RBCs (Fig. 1a). By contrast, FH‐RBCs with low LDL‐c (<2.5 mM) did not affect EDR (Fig. 1a). No differences were observed in EIDR between vessel segments exposed to FH‐RBCs with high LDL‐c compared to H‐RBCs (Fig. 1b), suggesting a selective and LDL‐c‐dependent impairment in endothelial function by FH‐RBCs. Based on this, the remaining mechanistic experiments were performed with RBCs from FH patients with LDL‐c >5.0 mM. In order to assess the influence of medication, subjects on versus off treatment at inclusion were compared (Fig. 1c). The magnitude of impairment in endothelial function induced by FH‐RBCs and LDL‐c >5.0 mM did not differ between those treated with lipid‐lowering medication and those free of medication at inclusion (Fig. 1c). The mean LDL‐c levels in these groups were comparable (6.8 mM in the group without medication and 7.2 mM in the group with medication). Subgroup analysis in the group with FH and LDL‐c >5.0 mM with regard to sex did not display any significant difference in RBC‐induced endothelial dysfunction between males and females (Fig. 1d).

### RBCs induce endothelial dysfunction by increasing vascular arginase

**Fig. 2:**
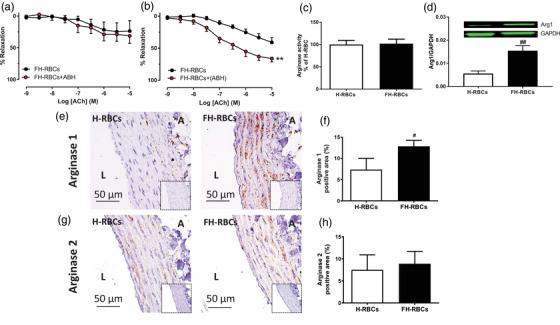
(a) Endothelium‐dependent relaxation (EDR) following incubation with red blood cells from patients with familial hypercholesterolemia (FH‐RBCs) (with low‐density lipoprotein cholesterol >5.0 mM) in the absence and presence of the arginase inhibitor 2(S)‐amino‐6‐boronohexanoic acid (ABH, 10 mM) throughout the 18‐h incubation (n = 6 each). (b) EDR following incubation with FH‐RBCs in the absence and presence of ABH (100 μM) only in the vessel following the 18‐h incubation (n = 6 each). (c) Arginase activity in RBCs from healthy subjects (H‐RBCs) and FH‐RBCs (n = 10 each). (d) Expression of arginase 1 with western blot (d, n = 6–9) or immunohistochemistry for arginase 1 (e, representative images; f, quantification of the positive areas, n = 6–7) or arginase 2 (g and h, n = 5 each) in aortic segments following 18‐h incubation with H‐RBCs and FH‐RBCs. A, adventitia; L, lumen. *p <0.01 versus FH‐RBCs; **p <0.05; ***p <0.01 vs. H‐RBCs, with matched two‐way analysis of variance (a and b), Student's t‐test (c), or Mann–Whitney test (d and f). Data are presented as mean and standard error of means.

To assess the possible influence of RBC arginase activity as a mechanism behind the induction of endothelial dysfunction, the arginase inhibitor ABH was added to the 18 h co‐incubation. This did not affect endothelial dysfunction induced by FH‐RBCs (Fig. 2a). By contrast, selective inhibition of vascular arginase attenuated the detrimental effect of FH‐RBCs and high LDL‐c on endothelial function (Fig. 2b). In line with this, no difference was observed in arginase activity between RBCs from FH patients and healthy subjects (Fig. 2c), whereas a marked increase in arginase 1 expression was observed in vascular segments incubated with FH‐RBCs (Fig. 2d). Using immunohistochemistry, it was revealed that FH‐RBCs but not H‐RBCs increased arginase 1 (Figs 2e,f) but not arginase 2 (Figs 2g,h) expression in endothelial cells and vascular smooth muscle cells. These observations suggest that vascular but not RBC arginase mediates the impairment in endothelial function induced by FH‐RBCs.

### ROS formation is increased in FH‐RBCs

**Fig. 3:**
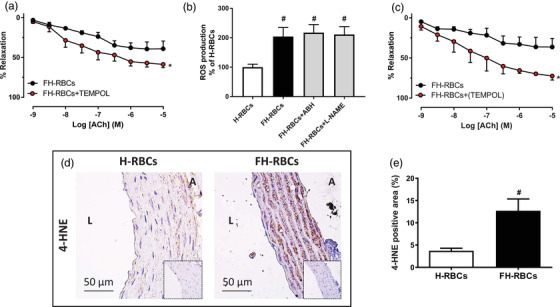
(a) Endothelium‐dependent relaxation following incubation with red blood cells from patients with familial hypercholesterolemia (FH‐RBCs) (with low‐density lipoprotein cholesterol >5.0 mM) in the absence and presence of the superoxide dismutase mimetic 4‐hydroxy‐2,2,6,6‐tetramethylpiperidine‐N‐oxyl (TEMPOL, 10 mM) throughout the 18‐h incubation (n = 5 each). (b) Production of reactive oxygen species (ROS) in RBCs from healthy subjects (H‐RBCs) and FH‐RBCs in the absence and presence of 2(S)‐amino‐6‐boronohexanoic acid (ABH) (1 mM) or the nitric oxide synthase inhibitor N^ω^‐nitro‐L‐arginine methyl ester (L‐NAME, 100 μM) (n = 8–10). (c) Presence of TEMPOL (100 μM) only in the vessel. Representative images of 4‐hydroxynonenal (HNE) in vascular segments following 18‐h incubation of H‐RBCs and FH‐RBCs (d) and quantification of positive areas (e, n = 6–7). *p <0.05 versus FH‐RBCs; **p <0.05 versus H‐RBCs, with matched two‐way analysis of variance (ANOVA) (a and c), one‐way ANOVA with Dunnett's multiple comparison test (b), or Mann–Whitney test (e). Data are presented as mean and standard error of means.

Next, we assessed the influence of superoxide by the application of the superoxide dismutase mimetic TEMPOL in the co‐incubation of RBCs and aortic segments. TEMPOL attenuated endothelial dysfunction induced by FH‐RBCs (Fig. 3a). This effect was in accordance with an increase in ROS production in FH‐RBCs in comparison with H‐RBCs (Fig. 3b). However, ROS production was not affected by ABH or the NOS inhibitor L‐NAME (Fig. 3b), which is in line with the observation that arginase activity did not differ between the groups (Fig. 2c). This suggests that sources other than eNOS arginase contribute to the altered redox balance in FH‐RBCs. Furthermore, the application of TEMPOL in the vasculature following incubation with FH‐RBCs completely rescued endothelial function (Fig. 3c). This was corroborated by the increased levels of the lipid peroxidation product 4‐HNE in the vasculature as a marker of oxidative stress (Figs 3d,e).

### Influence of antioxidants on RBC‐induced endothelial dysfunction

**Fig. 4:**
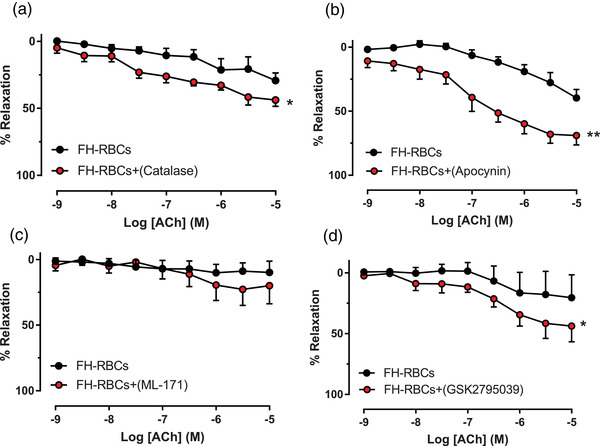
Endothelium‐dependent relaxation following incubation with red blood cells from patients with familial hypercholesterolemia (FH‐RBCs) (with low‐density lipoprotein cholesterol >5.0 mM) in the absence and presence of (a) hydrogen peroxide catalyst catalase (200 U/ml), (b) the NOX1/NOX2 inhibitor apocynin (100 μM), (c) NOX1 inhibitor 2‐acetylphenothiazine (ML‐171, 1 μM), and (d) NOX2 inhibitor N‐(1‐isopropyl‐3‐(1‐methylindolin‐6‐yl)‐1H‐pyrrolo[2,3‐b]pyridin‐4‐yl)‐1‐methyl‐1H‐pyrazole‐3‐sulfonamide (GSK2795039, 10 μM) (n = 3–6 in each panel). *p <0.05; **p <0.01 versus FH‐RBCs with matched two‐way analysis of variance (a–d). Data are presented as mean and standard error of means.

To further elucidate the pro‐oxidative effects of RBCs on the vascular wall, various antioxidants and scavengers were incubated in the organ chamber following the 18 h co‐incubation of FH‐RBCs and aortic segments with subsequent evaluation of EDR. These experiments revealed a significant improvement in the presence of the hydrogen peroxide catalyst catalase (Fig. 4a). Furthermore, we investigated whether NOXs represent a possible cause of endothelial dysfunction induced by RBCs. A complete rescue of endothelial function induced by FH‐RBCs was observed in the presence of the nonselective NOX1/2 inhibitor apocynin (Fig. 4b). To further distinguish which of the two isoforms plays an important role, a NOX1 (ML‐171, Fig. 4c) and a NOX2 inhibitor (GSK2795039, Fig. 4d) were administered in separate experiments. Only the NOX2 inhibitor significantly attenuated the degree of endothelial dysfunction induced by FH‐RBCs. This suggests that FH‐RBCs induce a NOX2‐dependent ROS formation in the vasculature, resulting in endothelial dysfunction.

### Lipidomic profiling in RBC lysates

**Fig. 5:**
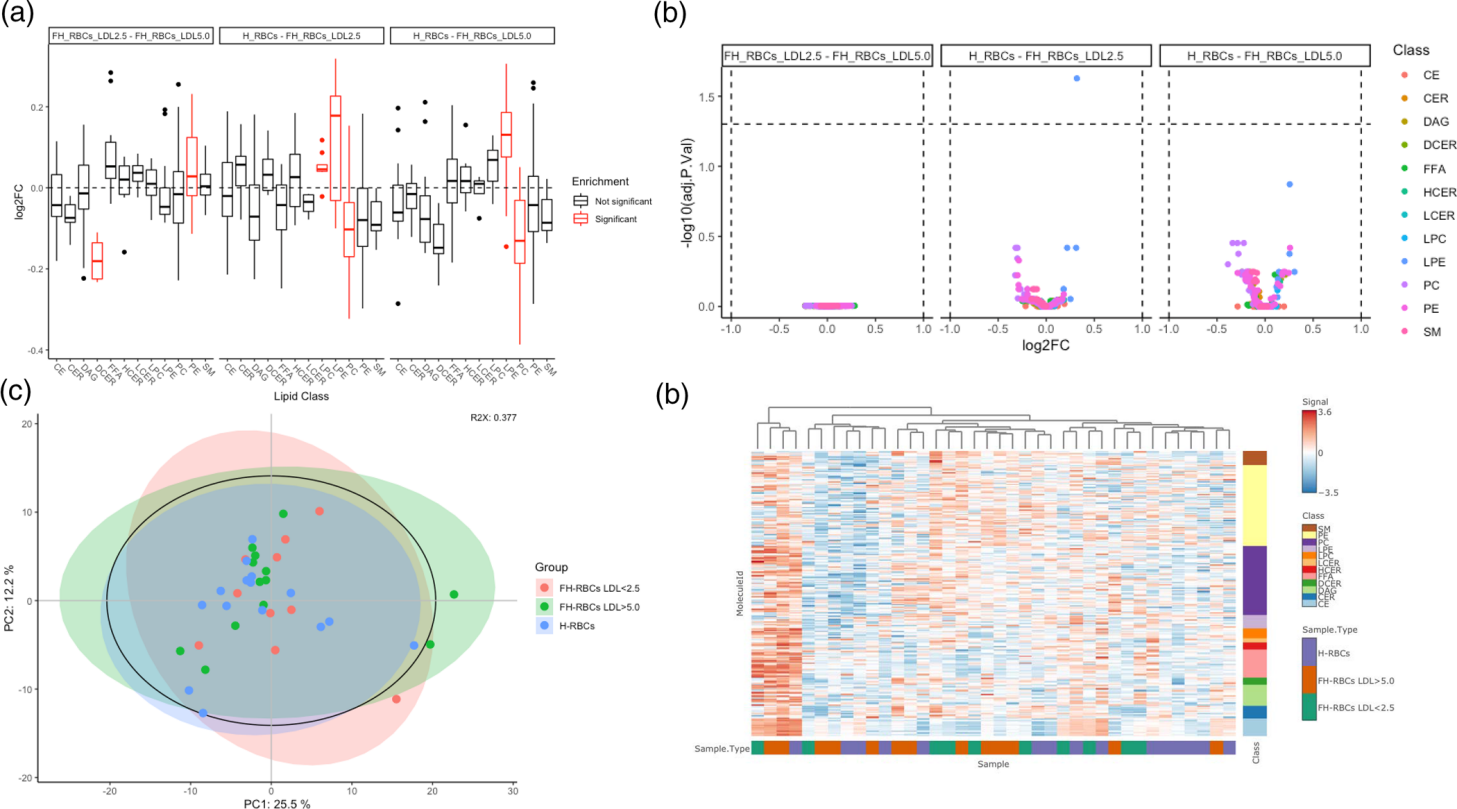
Visualization of the lipidomic analysis in red blood cell (RBC) lysates. (a) Lipid set enrichment analysis analyzing the distribution of lipid classes. Significantly enriched classes are marked in red. (b) Interactive volcano plot, with individual lipids as individual dots, colored by lipid class. (c) Principal component analysis plot colored by sample group. (d) An interactive heat map with hierarchical clustering of samples (x‐axis). CE, cholesteryl ester; CER, ceramide; DAG, diacylglycerol; DCER, dihydroceramide; FFA, free fatty acids; HCER, hexosylceramide; LCER, lactosylceramide; LPC, lysophosphatidylcholine; LPE, lysophosphatidylethanolamine; PC, phosphatidylcholine; PE, phosphatidylethanolamine; SM, sphingomyelin.

To thoroughly characterize the lipidome of the H‐RBCs and patients with FH and high or low LDL‐c levels, we performed a lipidomic profiling in RBC lysates. The major lipid classes in the RBCs are PC, PE, and SM (>90% of the total lipid content) (Table 2) [19]. However, the distribution across the study groups did not differ after adjusting for a false discovery rate. We further performed a lipid set enrichment analysis (LSEA), a method mirroring gene set enrichment analysis, used to detect preferential regulation of certain lipid classes with enrichment scores and significance calculated using an efficient permutation algorithm [16]. The LSEA revealed a difference in the composition of PC and LPE being significantly altered in both types of FH‐RBCs compared to H‐RBCs (Fig. 5a). Furthermore, a comparison between FH‐RBCs with LDL‐c <2.5 mM and FH‐RBCs with LDL‐c >5.0 mM revealed a significant alteration in DCER and PE compositions. However, the difference between the groups was small when considering the log fold change (Fig. 5a).

**Table 2:** Distribution of the lipid composition in red blood cell (RBC) lysates

	Group			Overall		H‐RBCs versus FH‐RBCs LDL >5.0		FH‐RBCs LDL <5.0 versus LDL <2.5		H‐RBCs versus FH‐RBCs LDL <2.5	
Variable	H‐RBCs (*n* = 15)	FH‐RBCs LDL >5.0 (*n* = 13)	FH‐RBCs LDL <2.5 (*n* = 10)	*p*‐Value	*p*‐Value adjusted	*p*‐Value	*p*‐Value adjusted	*p*‐Value	*p*‐Value adjusted	*p*‐Value	*p*‐Value adjusted
CE	0.80 (0.20, 7.07)	1.91 (0.87, 3.72)	1.11 (0.78, 1.50)	0.652	0.848	0.534	0.908	0.321	0.696	0.618	0.756
CER	0.76 (0.64, 0.83)	0.81 (0.70, 0.91)	0.85 (0.72, 1.00)	0.333	0.619	0.322	0.837	0.710	0.923	0.149	0.388
DAG	0.24 (0.18, 0.26)	0.22 (0.20, 0.25)	0.20 (0.18, 0.25)	0.850	0.987	0.872	0.908	0.577	0.923	0.657	0.756
DCER	0.08 (0.07, 0.10)	0.09 (0.08, 0.09)	0.10 (0.09, 0.11)	0.155	0.403	0.800	0.908	0.094	0.408	0.086	0.278
FFA	2.19 (1.83, 2.70)	2.17 (2.03, 2.21)	2.29 (1.95, 2.53)	0.987	0.987	0.908	0.908	0.901	0.951	0.912	0.912
HCER	0.06 (0.05, 0.07)	0.06 (0.05, 0.06)	0.05 (0.05, 0.06)	0.545	0.787	0.800	0.908	0.292	0.696	0.375	0.609
LCER	0.24 (0.21, 0.26)	0.26 (0.23, 0.27)	0.25 (0.22, 0.29)	0.391	0.635	0.174	0.566	0.951	0.951	0.375	0.609
LPC	1.00 (0.93, 1.12)	0.94 (0.86, 1.12)	0.75 (0.62, 0.82)	0.035	0.113	0.662	0.908	0.047	0.307	0.013	0.064
LPE	0.12 (0.10, 0.12)	0.13 (0.12, 0.14)	0.11 (0.10, 0.11)	0.014	0.060	0.029	0.124	0.006	0.082	0.437	0.632
PC	29.74 (28.95, 31.17)	28.01 (26.99, 28.19)	27.95 (27.05, 28.06)	0.011	0.060	0.009	0.120	0.664	0.923	0.015	0.064
PE	39.00 (36.76, 40.02)	40.40 (39.05, 42.83)	41.53 (40.14, 43.26)	0.011	0.060	0.025	0.124	0.385	0.715	0.007	0.064
SM	24.58 (23.14, 25.15)	24.31 (23.31, 24.78)	24.27 (22.97, 24.92)	0.924	0.987	0.800	0.908	0.901	0.951	0.698	0.756
TAG	0.20 (0.11, 1.05)	0.37 (0.20, 0.59)	0.48 (0.33, 0.68)	0.321	0.619	0.534	0.908	0.239	0.696	0.183	0.397

*Note*: Total lipid composition is expressed as the median percentage of total lipid content and Q1–Q3. *P*‐values were calculated with the nonparametric Kruskal–Wallis test (overall) and Mann–Whitney U for pairwise comparisons. The level of significance was set to 5%, two sided. The false discovery rate adjustment for multiple comparisons was applied within each *p*‐value group (column) (*p*‐value adjusted in tables). All analyses were performed using R version 4.0.2 (2020‐06‐22).

Abbreviations: CE, cholesteryl ester; CER, ceramide; DAG, diacylglycerol; DCER, dihydroceramide; FFA, free fatty acids; FH, familial hypercholesterolemia; HCER, hexosylceramide; H‐RBC, RBCs from healthy subjects; LCER, lactosylceramide; LPC, lysophosphatidylcholine; LDL, low‐density lipoprotein; LPE, lysophosphatidylethanolamine; PC, phosphatidylcholine; PE, phosphatidylethanolamine; SM, sphingomyelin; TAG, triacylglyceride.

Next, we analyzed the subspecies of the lipids using interactive volcano plots displaying the differences between the groups (Fig. 5b). Overall, this analysis revealed that only one lipid was significantly changed (LPE 20:4, *p* <0.05). Thus, although the LSEA indicated difference on a group level, there is little or no difference when analyzing the subspecies of the lipids. We also performed principal component analysis of the lipidomic dataset, which led to no visible clustering of the three groups, further indicating little variation (Fig. 5c). Finally, an interactive heatmap with hierarchical clustering of the samples on the x‐axis also supported the lack of alterations in the lipidome of the different groups, as the clustering resulted in clusters with a mixture of the sample types (Fig. 5d). Hence, the RBC lipidomes from the different groups showed small variations in the level of lipid classes, a variation that largely disappeared when further dissecting the data, with unsupervised clustering methods failing to cluster the samples according to the sample types. This collectively suggests that the lipid profile is not significantly altered in H‐RBCs compared to patients with FH, regardless of LDL‐c levels. All analyzed classes of lipids can be found in Supplementary file 1.

**Fig. 6:**
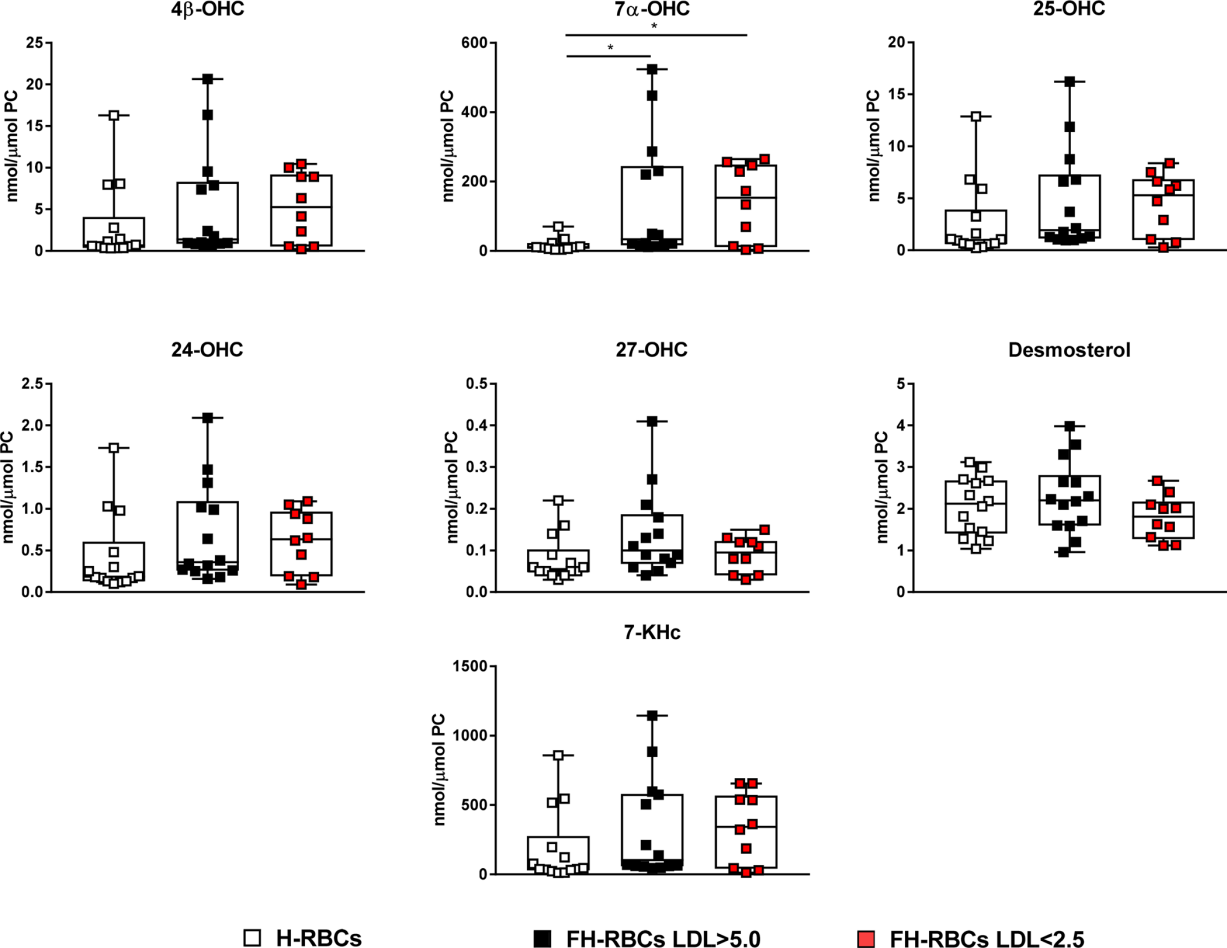
Quantification of the oxidized cholesterol products (oxysterols): 4β, 7α, 24, 25, and 27‐hydroxycholesterol (OHC), desmosterol, and 7‐ketocholesterol (7‐KCh). Data are presented as dot plots and boxplots (min.–max.) and expressed as nmol/μmol phosphatidylcholine (PC). *p <0.05 with Kruskal–Wallis and Dunn's post‐hoc test.

As RBC oxysterols, more specifically 7‐ketocholesterol, have been implicated as a source of ROS and a pathophysiological mediator in heart failure [20], we further aimed to dissect the source of increased ROS in RBCs from FH patients and elevated LDL‐c. Therefore, we quantified the oxysterol contents in RBC lysates, which revealed that only 7α‐hydroxycholesterol (OHC) was elevated in RBCs from both groups of FH compared to healthy subjects (Fig. 6). This might represent one of the sources of ROS in FH‐RBCs and implicates a possible effect of FH, independent of LDL‐c levels, on the oxysterol content in RBCs.

## Discussion

Hypercholesterolemia, a key risk factor for the development of atherosclerotic CVD, is known to cause functional and structural alterations of RBC properties [5]. Alterations in the RBC redox balance are predictive of future cardiovascular events in a high‐risk population [21], with unknown mechanisms. Here we show that RBCs isolated from well‐characterized patients with FH and elevated levels of LDL‐c induce impairment in endothelial function through increased ROS production. Consequently, by inhibition of RBC or vascular ROS, this impairment was almost completely prevented. Furthermore, FH‐RBCs and high LDL‐c (>5.0 mM) but not low LDL‐c (<2.5 mM) caused upregulation of vascular arginase 1. In line with this, inhibition of vascular arginase prevented endothelial dysfunction induced by FH‐RBCs and high LDL‐c. These data suggest that RBCs impair endothelial function in FH via an LDL‐dependent mechanism.

RBCs are known to be lipid storage, and there is evidence that the RBC is equipped with unique lipid transporters allowing certain lipids to reach the circulation [22]. RBCs are also known to incorporate cholesterol into the membranes and contribute to the progression of atherosclerotic plaque. Indeed, the cholesterol content in the RBC membrane is elevated in patients with acute coronary syndrome and vulnerable plaques [8]. Furthermore, high cholesterol decreases RBC deformability, which might lead to impaired capacity of oxygen delivery [7, 23]. It is also known that increased RBC membrane cholesterol attenuates ATP release, an effect that is reversed by statin treatment [24]. Statin treatment is also able to increase RBC deformability in hypercholesterolemia [23]. Contrary to these observations, the increased adhesiveness of RBCs from hypercholesterolemic patients to endothelial cells persists when hypercholesterolemic patients are treated with statins [25]. Collectively, these observations suggest that hypercholesterolemia drives RBC dysfunction and that some, but not all, alterations can be reversed by statins. In the present study, we provide an important extension of these observations by demonstrating that RBCs from patients with elevated LDL‐c induce impairment of endothelial function.

We observed that only FH‐RBCs and high LDL‐c but not those with low LDL‐c induced impairment of endothelial function. Since all patients with FH and low LDL‐c levels were treated with lipid‐lowering drugs, we aimed to dissect whether it is the treatment per se or the LDL‐c levels that explain the finding that FH‐RBCs with low LDL‐c did not impair endothelial function. FH‐RBCs with high LDL‐c levels with and without lipid‐lowering treatment induced a similar extent of impairment in endothelial function. This suggests that it is not the FH genotype per se nor the lipid‐lowering treatment, but rather the levels of LDL‐c that induce RBC dysfunction and subsequently endothelial dysfunction.

Previous studies have shown that increased ROS formation by RBCs is a central mechanism behind RBC‐induced endothelial dysfunction [5]. In line with this, we observed that ROS production was markedly elevated in FH‐RBCs. The source of ROS does not seem to be uncoupled eNOS or driven by elevated arginase activity, which is different from what has been observed in RBCs from patients with type 2 diabetes [9] and therefore remains elusive. It is known that a decrease in the RBC antioxidant capacity is predictive of future cardiovascular events [21]. This observation suggests that a shift in the redox balance in the RBC towards an increase in oxidative stress is transferred into cardiovascular injury. Indeed, we were able to demonstrate that FH‐RBCs and elevated LDL‐c impair endothelial function by inducing oxidative stress in the vasculature. Our functional data suggest that the main species affected in the vascular wall by FH‐RBCs are hydrogen peroxide and superoxide, which are likely enzymatically formed by eNOS uncoupling, but also NOX2. The increased oxidative stress in the endothelium is known to be a trigger for arginase activity [12]. Accordingly, we provide evidence that FH‐RBCs and high LDL‐c levels upregulate arginase 1 in the vasculature and consequently induce endothelial dysfunction. This mechanism may also be a source of superoxide production by uncoupling of eNOS due to L‐arginine depletion by arginase. It may therefore be reasonable to speculate that targeting the increase in RBC ROS or vascular arginase in patients with FH and hypercholesterolemia might be an attractive pharmacological intervention to alleviate cardiovascular complications in this patient group.

We have previously shown that RBCs in patients with type 2 diabetes also induce endothelial dysfunction [9]. It should be noted that there are some differences between patients with type 2 diabetes and FH regarding the mechanism by which the RBCs affect endothelial function. In the current study, we observed an increase in ROS production but not arginase activity in FH‐RBCs and LDL‐c >5.0 mM, and this increase was not attenuated by either arginase or NOS inhibition, which is the case in RBCs in type 2 diabetes. It is therefore unlikely that arginase‐induced eNOS uncoupling constitutes a major source of ROS production in the RBCs in FH. However, in line with type 2 diabetes [9] and COVID‐19 patients [10], RBCs from the current patient population induced an increase in vascular oxidative stress. The reason for the disparities between the patient groups remains unclear but it might be that LDL‐c and metabolic characteristics of type 2 diabetes might drive different mechanisms in the RBC.

Based on the above, we performed a lipidomic profiling on RBC lysates to dissect whether certain lipids are altered in patients with FH. However, no major alterations that distinguished the group of FH with LDL‐c >5.0 mM from the other groups were identified. This indicates that high levels of plasma LDL‐c do not significantly affect the lipid composition or the subclasses of lipid species in the RBCs. We further speculated that the increased ROS production in FH‐RBCs might be driven by higher levels of oxidized cholesterols (oxysterols). We observed that 7α‐OHC was increased in both groups of FH. It might therefore be reasonable to conclude that 7α‐OHC represents one of the sources of ROS in FH‐RBCs. However, based on the observation that only FH‐RBCs and LDL‐c >5.0 mM but not from LDL‐c <2.5 mM impair endothelial function, the elevation of 7α‐OHC does not explain the impairment in endothelial function induced by FH‐RBCs. This collectively implies that LDL‐c in the plasma drives increased oxidative stress via a mechanism that is independent of the lipid content and oxysterols in the RBCs.

Our data shed light on a previously overlooked pathogenic mechanism by which RBCs impair endothelial function. Since endothelial dysfunction is an early event in atherogenesis [26], it may be speculated that the RBC‐induced endothelial dysfunction demonstrated in the present study is of importance for the development of atherosclerosis among patients with FH and high LDL‐c. Importantly, the magnitude of impairment of endothelial function observed in patients with high LDL‐c levels despite medication with lipid‐lowering drugs is comparable to that observed with RBCs from treatment‐naïve patients. This reinforces the importance of aggressive LDL‐c management to normalize RBC function in patients with FH. Targeting RBC dysfunction in circumstances where this is not possible might represent a future therapeutic option.

This study has certain limitations. There is a slight difference in age and sex distribution across the groups. However, a younger population would possibly counteract the impairment in endothelial function induced by RBCs, which was not observed. Also, subgroup analysis of sex in the group with FH and high LDL‐c did not differ, and the sex distribution did not differ between the groups of FH with high and low LDL‐c but displayed a marked difference in RBC‐induced impairment in endothelial function. This collectively suggests that sex is not the sole explanation for the differences in FH‐RBC‐induced endothelial dysfunction in the current study. The ex vivo nature of the current study limits the extrapolation of the role of RBCs to the in vivo situation. However, only an ex vivo model allows the investigation of the effect of a single cell type, such as the RBC, on vascular function. It may also be argued that the use of different species (vessels from rats and RBCs from humans) might distort the results. However, we have previously shown that RBCs from patients with type 2 diabetes induce a comparable magnitude of impairment in endothelial function when using rat aortic rings and vascular rings from internal mammary arteries isolated from human subjects undergoing coronary artery bypass graft surgery [9]. This strongly suggests that the use of nonhuman vascular segments does not influence the results.

Moreover, there are additional unanswered questions that warrant further investigations. First, the source of ROS in RBCs is unclear as our data suggest that it is not derived from eNOS uncoupling. Furthermore, how the RBCs communicate with the endothelial cell lining and how NOX‐2 is activated in the vasculature following incubation with FH‐RBCs and elevated LDL‐c remain unknown. Considering the cell‐free zone between RBCs and the vasculature, there are multiple possible explanations for this. Export of signaling molecules may be through one of several transport protein channels of the RBC [27]. It was recently described that certain lipids are transported through a specific channel that is vital for maintaining RBC morphology [16]. These transporters may be altered in patients with FH, which deserves further attention in future investigations. An attractive alternative might be represented by the release of extracellular vesicles as these biological carriers are known to be enriched in RBCs [28]. Extracellular vesicles carrying signaling molecules are involved in the development of atherosclerosis [29]. A simple explanation would be that oxidative metabolites diffuse from the RBC membrane to resident cell types in the vasculature, which is unlikely considering that specifically targeting RBC ROS did not rescue endothelial dysfunction induced by FH‐RBCs and high LDL‐c. How plasma levels of LDL‐c affect RBC function, structure, and intracellular redox balance to become more pro‐oxidative certainly also deserves attention in future experimental investigations.

In conclusion, the present study demonstrates that FH‐RBCs and high LDL‐c induce endothelial dysfunction via an ROS‐mediated effect. Our data propose that RBCs may initiate a pathological sequence by a shift in the redox balance, consequently impairing endothelial function as an initial atherogenic step among patients with hypercholesterolemia.

The patient coordination by Ewa Steninger and Viveca Åberg and the technical assistance of Marita Wallin and David Ersgård are gratefully acknowledged. The manuscript has been handled by an external editor: Professor Jan Borén, Department of Molecular and Clinical Medicine at Institute of Medicine, University of Gothenburg, Sweden.

## Funding

The study was supported by the Swedish Heart and Lung Foundation (20190267 to A.M., 20190266 to J.P., and 20190341 and 20200326 to Z.Z.), the Swedish Research Council (2020‐01372 to J.P.), the Diabetes Research Wellness Foundation (720‐1519‐16 and 363‐PG to J.P.), the Stockholm County Council ALF (20190031 to J.P. and 20180512 to T.W.). O.K. is supported by the Swedish Society for Medical Research, SSMF (P19‐0068). A.I.C. is supported by a Novo Nordisk postdoctoral fellowship run in partnership with Karolinska Institutet.

## Conflict of interest

J.B. reports research grants from Akcea Therapeutics, Amgen, and Sanofi not relevant to the content of the current study. The remaining authors declare no conflict of interest.

## Author contributions

Ali Mahdi: Conceptualization; data curation; formal analysis; investigation; methodology; visualization; writing – original draft; writing – review and editing. Tigist Wodaje: Conceptualization; data curation; formal analysis; writing – original draft; writing – review and editing. Oskar Kövamees: Conceptualization; formal analysis; writing – review and editing. John Tengbom: Data curation; investigation; methodology; writing – review and editing. Allan Zhao: Formal analysis; methodology; software; visualization; writing – original draft; writing – review and editing. Tong Jiao: Data curation; methodology; writing – review and editing. Marcus Henricsson: Conceptualization; formal analysis; investigation; methodology; writing – review and editing. Jiangning Yang: Conceptualization; investigation; methodology; supervision; writing – review and editing. Zhichao Zhou: Conceptualization; data curation; formal analysis; investigation; methodology; project administration; supervision; writing – review and editing. Anna I. Nieminen: Data curation; methodology; writing – review and editing. Malin Levin: Conceptualization; funding acquisition; resources; writing – review and editing. Aida Collado: Conceptualization; data curation; formal analysis; investigation; methodology; writing – review and editing. Jonas Brinck: Conceptualization; project administration; resources; supervision; writing – original draft; writing – review and editing. John Pernow: Conceptualization; data curation; formal analysis; funding acquisition; investigation; methodology; project administration; resources; supervision; visualization; writing – original draft; writing – review and editing.

## Supporting information


**Supplementary File 1**. All analyzed classes of lipids.

## References

[1] Michos ED , McEvoy JW , Blumenthal RS . Lipid management for the prevention of atherosclerotic cardiovascular disease. N Engl J Med. 2019;381:1557–67. 31618541 10.1056/NEJMra1806939

[2] Defesche JC , Gidding SS , Harada‐Shiba M , Hegele RA , Santos RD , Wierzbicki AS . Familial hypercholesterolaemia. Nat Rev Dis Primers. 2017;3:17093. 29219151 10.1038/nrdp.2017.93

[3] EAS Familial Hypercholesterolaemia Studies Collaboration (FHSC) . Global perspective of familial hypercholesterolaemia: a cross‐sectional study from the EAS Familial Hypercholesterolaemia Studies Collaboration (FHSC). Lancet. 2021;398:1713–25. 34506743 10.1016/S0140-6736(21)01122-3

[4] Libby P , Ridker PM , Hansson GK , Leducq Transatlantic Network on Atherothrombosis . Inflammation in atherosclerosis: from pathophysiology to practice. J Am Coll Cardiol. 2009;54:2129–38. 19942084 10.1016/j.jacc.2009.09.009 PMC2834169

[5] Mahdi A , Cortese‐Krott MM , Kelm M , Li N , Pernow J . Novel perspectives on redox signaling in red blood cells and platelets in cardiovascular disease. Free Radic Biol Med. 2021;168:95–109. 33789125 10.1016/j.freeradbiomed.2021.03.020

[6] Pernow J , Mahdi A , Yang J , Zhou Z . Red blood cell dysfunction: a new player in cardiovascular disease. Cardiovasc Res. 2019;115:1596–605. 31198931 10.1093/cvr/cvz156

[7] Kanakaraj P , Singh M . Influence of hypercholesterolemia on morphological and rheological characteristics of erythrocytes. Atherosclerosis. 1989;76:209–18. 2730718 10.1016/0021-9150(89)90105-6

[8] Tziakas DN , Kaski JC , Chalikias GK , Romero C , Fredericks S , Tentes IK , et al. Total cholesterol content of erythrocyte membranes is increased in patients with acute coronary syndrome: a new marker of clinical instability? J Am Coll Cardiol. 2007;49:2081–9. 17531656 10.1016/j.jacc.2006.08.069

[9] Zhou Z , Mahdi A , Tratsiakovich Y , Zahorán S , Kövamees O , Nordin F , et al. Erythrocytes from patients with type 2 diabetes induce endothelial dysfunction via arginase I. J Am Coll Cardiol. 2018;72:769–80. 30092954 10.1016/j.jacc.2018.05.052

[10] Mahdi A , Collado A , Tengbom J , Jiao T , Wodaje T , Johansson N , et al. Erythrocytes induce vascular dysfunction in COVID‐19. JACC Basic Transl Sci. 2022;7(3):193–204. 35194565 10.1016/j.jacbts.2021.12.003 PMC8849181

[11] Zhou Z , Collado A , Sun C , Tratsiakovich Y , Mahdi A , Winter H , et al. Downregulation of erythrocyte miR‐210 induces endothelial dysfunction in type 2 diabetes. Diabetes. 2022;71:285–97. 34753800 10.2337/db21-0093

[12] Mahdi A , Kovamees O , Pernow J . Improvement in endothelial function in cardiovascular disease—is arginase the target? Int J Cardiol. 2020;301:207–14. 31785959 10.1016/j.ijcard.2019.11.004

[13] Yang J , Gonon AT , Sjoquist PO , Lundberg JO , Pernow J . Arginase regulates red blood cell nitric oxide synthase and export of cardioprotective nitric oxide bioactivity. Proc Natl Acad Sci U S A. 2013;110:15049–54. 23980179 10.1073/pnas.1307058110 PMC3773799

[14] Yang J , Zheng X , Mahdi A , Zhou Z , Tratsiakovich Y , Jiao T , et al. Red blood cells in type 2 diabetes impair cardiac post‐ischemic recovery through an arginase‐dependent modulation of nitric oxide synthase and reactive oxygen species. JACC Basic Transl Sci. 2018;3:450–63. 30175269 10.1016/j.jacbts.2018.03.006 PMC6115643

[15] Ghorasaini M , Mohammed Y , Adamski J , Bettcher L , Bowden JA , Cabruja M , et al. Cross‐laboratory standardisation of preclinical lipidomics using differential mobility spectrometry and multiple reaction monitoring. Anal Chem. 2021;93:16369–78. 34859676 10.1021/acs.analchem.1c02826 PMC8674878

[16] Mohamed A , Molendijk J , Hill MM . lipidr: a software tool for data mining and analysis of lipidomics datasets. J Proteome Res. 2020;19:2890–7. 32168452 10.1021/acs.jproteome.0c00082

[17] Penkert H , Lauber C , Gerl MJ , Klose C , Damm M , Fitzner D , et al. Plasma lipidomics of monozygotic twins discordant for multiple sclerosis. Ann Clin Transl Neurol. 2020;7:2461–6. 33159711 10.1002/acn3.51216 PMC7732246

[18] Honda A , Yamashita K , Hara T , Ikegami T , Miyazaki T , Shirai M , et al. Highly sensitive quantification of key regulatory oxysterols in biological samples by LC‐ESI‐MS/MS. J Lipid Res. 2009;50:350–7. 18815436 10.1194/jlr.D800040-JLR200

[19] Aoun M , Corsetto PA , Nugue G , Montorfano G , Ciusani E , Crouzier D , et al. Changes in red blood cell membrane lipid composition: a new perspective into the pathogenesis of PKAN. Mol Genet Metab. 2017;121:180–9. 28456385 10.1016/j.ymgme.2017.04.006

[20] Tang H‐Y , Wang C‐H , Ho H‐Y , Wu P‐T , Hung C‐L , Huang C‐Y , et al. Lipidomics reveals accumulation of the oxidised cholesterol in erythrocytes of heart failure patients. Redox Biol. 2018;14:499–508. 29101899 10.1016/j.redox.2017.10.020 PMC5675899

[21] Blankenberg S , Rupprecht HJ , Bickel C , Torzewski M , Hafner G , Tiret L , et al. Glutathione peroxidase 1 activity and cardiovascular events in patients with coronary artery disease. N Engl J Med. 2003;349:1605–13. 14573732 10.1056/NEJMoa030535

[22] Vu TM , Ishizu AN , Foo JC , Toh XR , Zhang F , Whee DM , et al. Mfsd2b is essential for the sphingosine‐1‐phosphate export in erythrocytes and platelets. Nature. 2017;550:524–8. 29045386 10.1038/nature24053

[23] Kohno M , Murakawa K , Yasunari K , Yokokawa K , Horio T , Kano H , et al. Improvement of erythrocyte deformability by cholesterol‐lowering therapy with pravastatin in hypercholesterolemic patients. Metabolism. 1997;46:287–91. 9054471 10.1016/s0026-0495(97)90255-9

[24] Forsyth AM , Braunmuller S , Wan J , Franke T , Stone HA . The effects of membrane cholesterol and simvastatin on red blood cell deformability and ATP release. Microvasc Res. 2012;83:347–51. 22349292 10.1016/j.mvr.2012.02.004

[25] Cilla A , López‐García G , Collado‐Díaz V , Amparo Blanch‐Ruiz M , Garcia‐Llatas G , et al. Hypercholesterolemic patients have higher eryptosis and erythrocyte adhesion to human endothelium independently of statin therapy. Int J Clin Pract. 2021;75:e14771. 34473881 10.1111/ijcp.14771

[26] Gimbrone MA Jr , Garcia‐Cardena G . Endothelial cell dysfunction and the pathobiology of atherosclerosis. Circ Res. 2016;118:620–36. 26892962 10.1161/CIRCRESAHA.115.306301 PMC4762052

[27] Pretini V , Koenen MH , Kaestner L , Fens M , Schiffelers RM , Bartels M , et al. Red blood cells: chasing interactions. Front Physiol. 2019;10:945. 31417415 10.3389/fphys.2019.00945 PMC6684843

[28] Thangaraju K , Neerukonda SN , Katneni U , Buehler PW . Extracellular vesicles from red blood cells and their evolving roles in health, coagulopathy and therapy. Int J Mol Sci. 2020;22:153. 33375718 10.3390/ijms22010153 PMC7796437

[29] Boulanger CM , Loyer X , Rautou PE , Amabile N . Extracellular vesicles in coronary artery disease. Nat Rev Cardiol. 2017;14:259–72. 28150804 10.1038/nrcardio.2017.7

